# Color polymorphism in the Cuban endemic livebearing fish *Limia vittata* (Teloestei, Poeciliidae): Potential roles of sexual and natural selection

**DOI:** 10.1002/ece3.9768

**Published:** 2023-01-24

**Authors:** Rodet Rodriguez‐Silva, Montrai Spikes, Manuel Iturriaga Monsisbay, Ingo Schlupp

**Affiliations:** ^1^ Department of Biology University of Oklahoma Norman Oklahoma USA; ^2^ Departamento de Colecciones Zoológicas Instituto de Ecología y Sistemática La Habana Cuba

**Keywords:** color polymorphism, *Limia*, mate choice, natural selection, poeciliid, salinity gradient, sexual selection

## Abstract

Color polymorphism can be maintained in natural populations by natural selection or sexual selection. In this study, we use two different approaches to test which of these evolutionary mechanisms may explain the presence of color polymorphism in the Cuban Limia (*Limia vittata*), an endemic livebearing fish from Cuba. First, we investigate the role of sexual selection using traditional binary choice tests looking at both female and male preferences relative to varying degrees of black spotting in stimulus mates. Second, we assess the role of natural selection by analyzing the frequency and geographic distribution of black‐spotted and nonspotted morphs of *L. vittata* in natural populations from Cuba. The frequency of black‐spotted morphs is significantly higher in brackish and saltwater environments compared with freshwater habitats, which could be related to higher predation pressure in coastal ecosystems compared with purely freshwater environments. Our results suggest that habitat variation is the most important factor in maintaining color polymorphism in *L. vittata*. Salinity levels could be indirectly responsible for maintaining different color morphs in this species, likely due to the regulatory effect of saline gradients on predation regimes.

## Graphical abstract

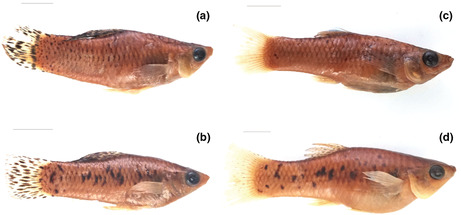

## 1 INTRODUCTION

Polymorphism can be defined as the occurrence of multiple discrete forms or morphs within populations of a species. This is a common phenomenon that can be caused and modulated by selection, drift, mutations, and gene flow (Charlesworth & Charlesworth, 1975; Guerrero & Hahn, 2017; Kang et al., 2017; Oxford, 2005; Rojas et al., 2020). As a special case of polymorphism, phenotypes can show discrete variations in color. Such color polymorphisms are common and widespread (Gray & McKinnon, 2007; White & Kemp, 2016), and have been well studied across several taxa, such as aposematic poison frogs of the genus *Dendrobates* (Hoogmoed & Avila‐Pires, 2012; Stuckert et al., 2019); and different species of lizards (Bleay & Sinervo, 2007; Vercken et al., 2007); Gouldian finches (*Erythrura gouldiae*) (Gilby et al., 2009), among many.

Theoretically, color polymorphism (and polymorphism in general) can be maintained by sexual selection or by natural selection acting separately (Gosden & Svensson, 2009; Hughes et al., 2013; Iserbyt et al., 2013) or by a combination of the two mechanisms. If, for example, natural selection was invoked, one would assume a benefit to a particular phenotype that, for example, is better suited to avoid predators (Punzalan et al., 2005). Often this is governed by frequency‐dependent selection, usually with morph variants that are rare, having a selective advantage. This phenomenon results in higher fitness that causes negative frequency‐dependent selection (NFDS), as shown in both classical and recent studies (Ayala & Campbell, 1974; Chouteau et al., 2017; Clarke & Donald, 1962; Ford, 1945; Heino et al., 1998; Kettlewell, 1961; Kurbalija et al., 2020; Svardal et al., 2015).

Conversely, one would assume sexual selection to maintain a polymorphism if, for example, females show preferences for one of several color phenotypes in males. In this case, a particular color morph influences mating interactions by changes in mating preferences, which alters morph‐specific fitness and also maintains genetic diversity (Hughes et al., 2013; Iserbyt et al., 2013; Roulin & Ducrest, 2013). Of course, negative frequency‐dependent selection may be the mechanism here, too. In some cases, however, neither natural selection nor sexual selection alone explains how polymorphisms are maintained and scenarios where both agents act simultaneously are more apt to explain the presence of color polymorphism (Wellenreuther et al., 2014). This has been shown in populations of guppies (*Poecilia reticulata*) where color morphs are exposed to strong sexual and natural selection as agents upholding the diversity in color patterns in males. Studies have shown that males of this species that exhibit rare color morphs have a reproductive advantage over males with common phenotypes (Hampton et al., 2009; Hughes et al., 2013; Olendorf et al., 2006).

In the family Poeciliidae, the general mating system is characterized by promiscuity, and mostly female choice, with male choice occasionally found. Males either court females or attempt to force copulations. Multiple paternity seems common. Males do not contribute to parental care beyond providing sperm but are often highly ornamented (Becker et al., 2012; Bisazza, 1993; Furness et al., 2020). Prominent members of this family include the guppy, swordtails, and mollies. The latter, in the genus *Poecilia*, is the sister taxon to the genus *Limia*.

The most common pattern of color polymorphism observed in natural conditions is both sexes exhibiting polymorphism within a population (Ford, 1945). For example, this is the case in the poeciliid fish species *Limia vittata*, which is endemic to the island of Cuba in the West Indies. The genus *Limia* is endemic to the Caribbean where it shows a notable radiation on the island of Hispaniola with 19 described species so far (Burgess & Franz, 1989; Chambers, 1987; Rodriguez‐Silva et al., 2020; Spikes et al., 2021; Weaver et al., 2016). However, *L. vittata* is the sole species in the genus found in Cuba, where it has a ubiquitous presence in lowland water bodies ranging from freshwater to brackish and hypersaline habitats (Barus et al., 1980; Ponce de León & Rodriguez, 2010). As is typical for fishes of this family, there is a prominent sexual dimorphism with the females being larger on average. In addition, this species shows a discrete color polymorphism in both sexes that varies in frequency with habitat. Both sexes have a varying number of black spots or can be completely unspotted. Other species in the same genus do not show any color polymorphism, but several other species in the family do (Zerulla & Stoddard, 2021). In addition, the occurrence of male‐limited color polymorphism is frequent in poeciliids in general (Hurtado‐Gonzalez et al., 2010, 2014; Lindholm et al., 2004, 2015; Zerulla & Stoddard, 2021). The fact that in *L. vittata* both sexes are polymorphic and that the variation in melanism occurs across populations allows us to ask what might maintain spotted and nonspotted phenotypes on a species, population, and individual level and to contrast natural and sexual selection as the underlying mechanism.

Therefore, in this study, we combined data from two binary choice experiments trying to elucidate a potential role of sexual selection with population‐based field survey data, potentially reflecting patterns of natural selection.

Concretely, we investigated the role of sexual selection in two mate choice studies, looking at both female and male mate choice. For this, we used black‐spotted aquarium stocks of *L. vittata*, which have likely been artificially selected by breeders to have heavily spotted individuals to determine whether there is a clear preference for more or less spotted mates. We used these stocks because we assume that if sexual selection can be detected it would likely be in a lineage that has been selected to have heavy spotting. In addition, it is currently not within our reach to collect fresh populations from Cuba. Furthermore, we investigated the role of habitat variation and natural selection in color polymorphism by comparing populations that naturally live in different types of waterbodies. To do this, we collected information about the frequency and geographic distribution of melanistic and nonmelanistic morphs of *L. vittata* of natural populations in Cuba. Thus, we tested the hypothesis that melanistic phenotypes are associated with habitats of different salinity. Together, we explore which mechanisms (sexual selection, natural selection, or a combination of both) may more likely contribute to the maintenance of the color polymorphism found in the Cuban Limia.

## 2 MATERIALS AND METHODS

### 2.1 Classification of fish samples based on the total body area covered by black spots (for the sexual selection experiment)

In this study, we used two populations of *L. vittata* obtained from randomly outbred stocks that are kept in common garden conditions at the OU International Stock Center for Livebearing Fishes in a greenhouse at the Aquatic Research Facility at the University of Oklahoma. Unfortunately, information on the collection sites and dates for these populations is not available and collections in Cuba are currently impossible. Prior to the experiment, all individuals were transported to an indoor fish laboratory with a temperature of 26°C (±3°C), where the individual fish were separated by sex and kept in 75 L tanks on a 12‐h day‐night cycle for at least 30 days acclimation. Fish were fed ad libitum twice a day with a mixed diet that included frozen bloodworms, *Daphnia*, brine shrimp nauplii, and TetraMin flakes.

**FIGURE 1:**
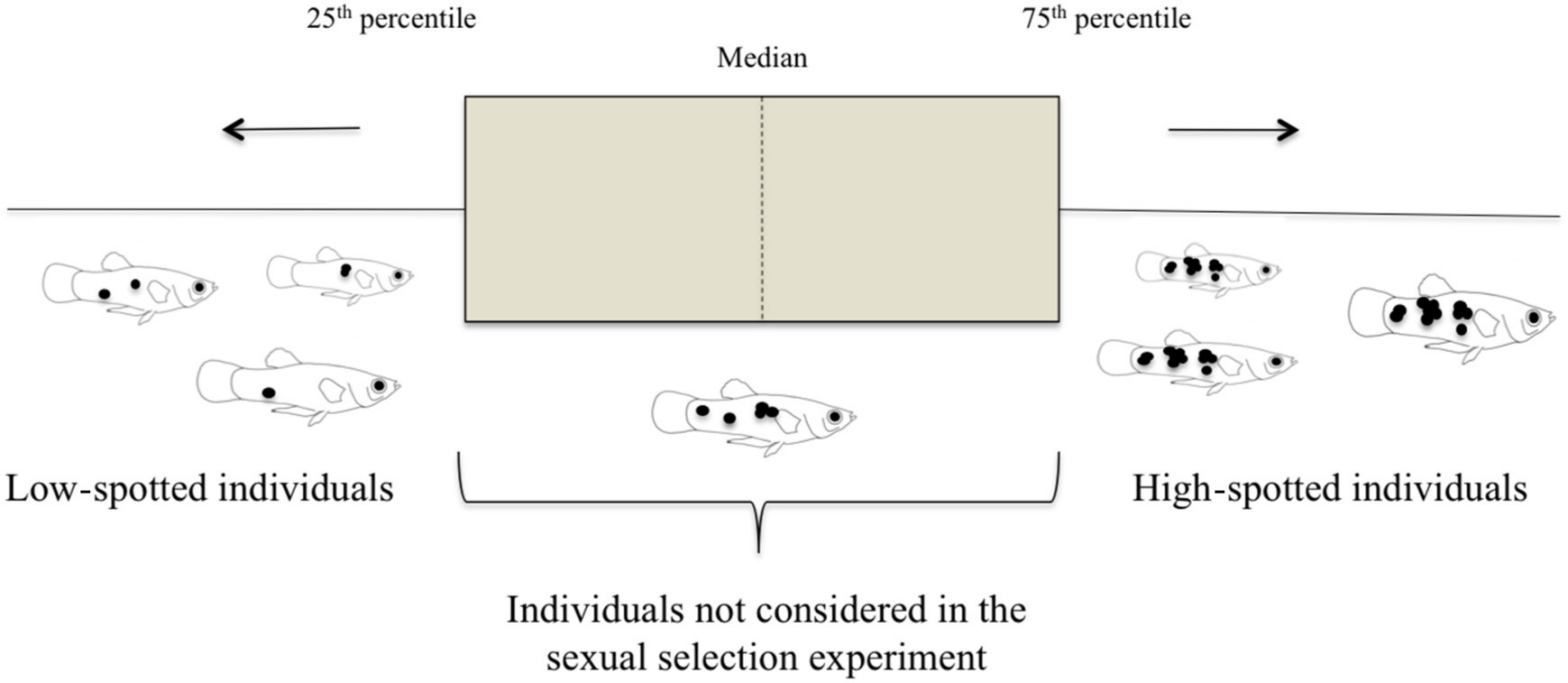
Classification of stimulus fish in the sexual selection experiment based on the total body area covered by black spots.

To measure the body area covered by black spots on each individual, 60 males and 80 females were anesthetized one at a time with buffered tricaine‐methanesulfonate (MS‐222) and photographed with a Nikon D5200 camera. We captured both lateral views. The photos were used to measure the standard length and total body area covered by black spots for each fish using ImageJ 1× software (Schneider et al., 2012). Then, we analyzed the distributions of the spot patterns of male and female individuals separately in our sample and split them into different categories depending on the number of spots per lateral view with both sides taken together. Individuals that fell in the lower quartile or 25th percentile (i.e., with a low spot density per surface area) were considered low‐spotted and the ones that fell in the upper quartile or 75th percentile (i.e., with higher spot density) were classified as highly spotted individuals (Figure 1). We only used stimulus fish in both treatments (male and female choice) that were similar in size (±1.5 mm) and included the size of stimuli fish as a covariate in our statistical analysis.

### 2.2 Sexual selection experiments

Dichotomous choice tests were conducted in a 75 L tank that was divided into three equal sections in length. To divide the tank, we drew two vertical lines on the front glass to delimit preference zones. Within each section was 8.5 cm × 8.5 cm × 46 cm clear, unperforated Plexiglas prism with rectangular shape. These containers were used to restrict movement, mechanosensory signals, and chemical cues, while still allowing visual communication of fish. The experimenter then sat 3 m away directly in front of the test tank and recorded the amount of time a focal fish spent within the outer sections (stimuli fish) of the tank. The three sections of the tank, demarcated as left choice zone, middle neutral zone, and right choice zone were of the same size (32 cm × 32 cm × 43 cm). In other experiments, we successfully used this method to detect preferences (Makowicz et al., 2016).

Because we wanted to examine both female and male choice, the tests began by placing focal a fish (male or female) in the Plexiglas container of the neutral zone of the tank. Then, a high‐spotted and a low‐spotted stimulus fish of the opposite sex were randomly placed on either side of the focal fish. Fish were then undisturbed for 5 min to acclimate to the test tank (habituation phase). After acclimation, the focal fish was released from the neutral zone to swim freely throughout the test tank for 5 min. We recorded the amount of time the focal fish spent within the section with either the high‐spotted or low‐spotted stimulus fish (association time). After the 5‐min trial, the focal fish was returned to the container in the neutral zone and the stimulus fish were switched to the opposite outer section of the tank. We performed a second trial with the stimulus fish swapped, to detect any side bias. We gave the fish (focal and stimuli) another 5 min of acclimation and then released the focal fish to record the association time for another 5 min. Once each trial was completed, we measured the standard length of the focal fish by placing it on a millimeter grid. Although we attempted to use different stimulus fish in every trial during the two dichotomous choice assessments (male preference and female preference), it was necessary to reuse some individuals to create sufficient numbers of stimulus pairs with contrasting spotted areas. However, no stimulus fish were reused more than once, and all of the reused individuals were tested in different dyadic combinations (Sommer‐Trembo et al., 2016). No focal fish were reused in the experiment.

We calculated the association time of the focal fish with each stimulus fish by adding the time the focal fish spent associated with each choice zone in the two parts of each trial separately in males and females, and then calculated the strength of preference (SOP) as: SOP = (time spent with more spotted stimulus − time spent with less spotted stimulus)/time spent with both stimuli. Thus, SOP values could range from +1 (maximum preference for the high‐spotted stimulus) to −1 (maximum preference for the low‐spotted stimulus). No preference would be indicated by SOP values around 0. We assessed female preference in 24 individuals with different male choices according to the spotted area (SA): low‐spotted males (mean spotted area ± SE: 9.02 ± 2.08 mm^2^) and high‐spotted males (mean spotted area ± SE: 21.66 ± 2.94 mm^2^) (t (7) = −9.91, *p* < .00001). In the case of males, we tested 25 individuals to determine any preference for less or more spotted females: low‐spotted females (mean spotted area ± SE: 19.94 ± 3.43 mm^2^) and high‐spotted females (mean ± SE (SA): 64.50 ± 13.12 mm^2^) (t (6) = −8.70, *p* < .00001).

### 2.3 Relation between frequency and geographic distribution in melanistic and nonmelanistic morphs of natural populations of *L. vittata*

**FIGURE 2:**
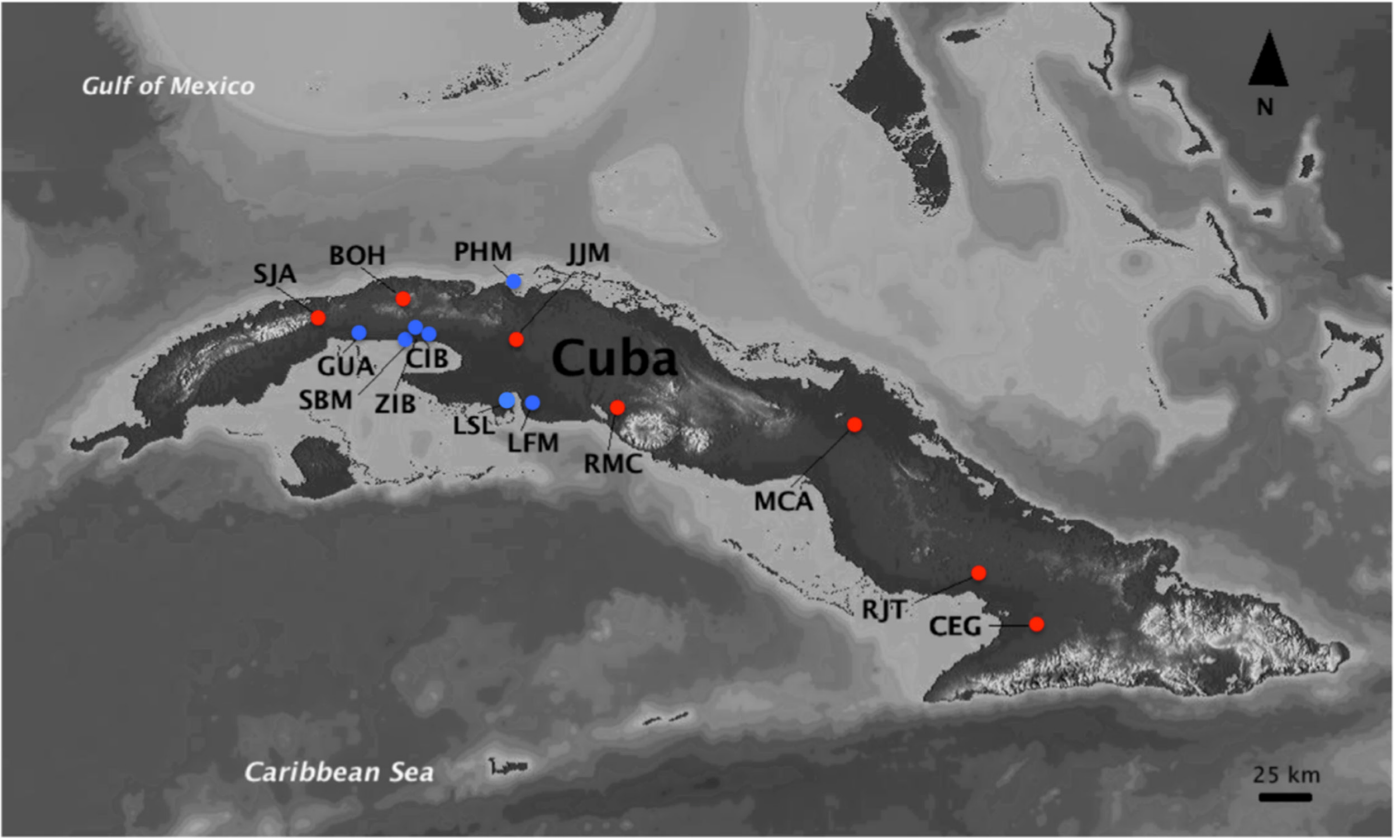
Geographic distribution of the field sites where *Limia vittata* specimens were collected to evaluate the natural occurrence of melanistic morphs. Locality codes are represented by three capital letters are described in Table 1. Red dots represent freshwater habitats and blue dots brackish or saltwater environments (Map source: StreetMap contributors).

We analyzed individuals of *L. vittata* collected by us from multiple localities in Cuba in 2018 and 2019, as well as voucher specimens from additional localities from the scientific collections at the Instituto de Ecología y Sistemática in Cuba (CZACC) (Figure 2; Table 1). Even though many physicochemical water parameters show similar ranges along the continuous distribution of *L. vittata* on the Cuban archipelago, this is not the case for salinity levels since the species can be found in freshwater, saline, and even hypersaline habitats. For this reason, we included populations from environments with contrasting salinities to evaluate whether this abiotic factor could be associated with different black‐spotted morph proportions. For coastal localities (represented by blue dots in Figure 2), salinity levels were confirmed either by direct measurements on site using a conductivity meter or refractometer (collections made in 2018 and 2019) or through personal communication with the collector Tec. J. Rodolfo Sánchez Correa.

**TABLE 1:** Number (M: males, F: females) of *L. vittata* specimens analyzed from multiple populations in Cuba. Populations were selected based on the association with habitats of different salinity levels. Locality codes are in parenthesis after the locality name with corresponding salinity values (when available).

Locality	Collection date and source of specimens	GPS coordinates	Melanistic individuals	Nonmelanistic individuals	Subtotal
Freshwater					
Río San Juan, Sierra del Rosario, Candelaria, Artemisa (SJA)	Apr/1982 CZACC collection	22.848217, −82.945286	3 F, 0 M	12 F, 4 M	19
Río San José, Jovellanos, Matanzas (JJM)	Sep/1982 CZACC collection	22.776520, −81.249290	1 F, 0 M	6 F, 3 M	10
Boyeros, Havana (BOH, 593 mS/cm)	Aug/2018 Field collection	23.045658, −82.370904	0 F, 0 M	13 F, 0 M	13
El Venero, Morón, Ciego de Ávila (MCA, 810 mS/cm)	Jan/2019 Field collection	22.075944, −78.496111	0 F, 0 M	17 F, 14 M	31
Cauto Embarcadero, Granma (CEG, 410 mS/cm)	Jan/2019 Field collection	20.624414, −76.928889	0 F, 0 M	6 F, 3 M	9
Río Jobabo, Las Tunas (RJT, 760 mS/cm)	Jan/2019 Field collection	20.881805, −77.300997	1 F, 0 M	6 F, 16 M	23
Río Matagua, Cumanayagua, Cienfuegos (RMC 336 mS/cm)	Jan/2019 Field collection	22.052714, −80.291944	0 F, 0 M	4 F, 1 M	5
Total			5 F, 0 M	64 F, 41 M	110
Saltwater					
Canal del Indio, Surgidero de Batabano, Mayabeque (CIB)	Nov/1984 CZACC collection	22.687590, −82.2268841	4 F, 2 M	28 F, 11 M	45
Zanja del Indio, Surgidero de Batabano, Mayabeque (ZIB)	May/1985 CZACC collection	22.691526, −82.268179	8 F, 1 M	67 F, 3 M	79
Surgidero de Batabano, Mayabeque (SBM)	Feb/1986 CZACC collection	22.685079, −82.290201	4 F, 6 M	16 F, 41 M	67
Laguna de Facundo, Ciénaga de Zapata, Matanzas (LFM, 14 ppt)	Mar/2014 Field collection	22.280148, −81.163460	3 F, 1 M	6 F, 10 M	20
Península de Hicacos, Matanzas (PHM, 35 ppt)	Nov/2015 Field collection	23.192912, −81.162099	4 F, 0 M	76 F, 7 M	87
Guanímar, Artemisa (GUA, 30 ppt)	Aug/2018 Field collection	22.694176, −82.651991	3 F, 2 M	19 F, 15 M	39
Las Salinas de Brito, Ciénaga de Zapata, Matanzas (LSL, 33 ppt)	Jan/2019 Field collection	22.177989, −81.253661	28 F, 2 M	49 F, 3 M	82
Total			54 F, 14 M	261 F, 90 M	419

**FIGURE 3:**
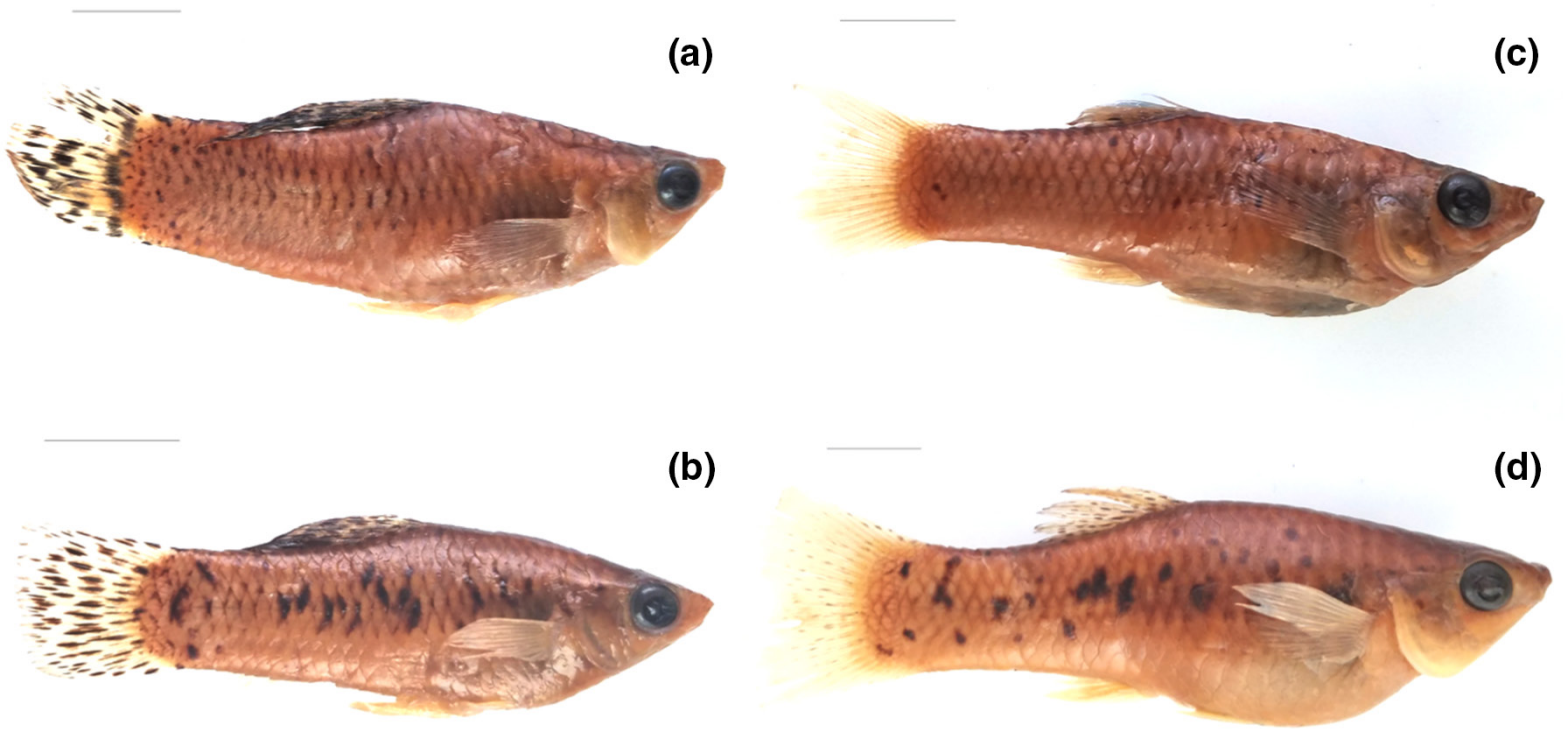
Photographs of nonmelanistic (top) and melanistic morphs (bottom) deposited in the collections of the Instituto de Ecología y Sistemática in Cuba: (a) nonmelanistic male (CZACC 9.07), (b) melanistic male (CZACC 9.01), (c) nonmelanistic female (CZACC 9.21), (d) melanistic female (CZACC 9.02).

We recorded the number of melanistic and nonmelanistic morphs by sex in each location (Table 1; Figure 3). In the case of melanistic individuals, we measured the body area covered by black spots or blotches on each fish by using the same procedure described for aquarium‐raised specimens used in the sexual selection experiment.

### 2.4 Statistical analysis

#### 2.4.1 Sexual selection experiment

Equal variances and normality of residuals were checked using a Levene's test and a Shapiro–Wilk's test, respectively, prior to inferential analyses, and both assumptions were met for our data. In our mate choice experiment, we predicted that sexual selection might explain the presence of color polymorphism in some populations of *L. vittata* and that either males or females would show a preference for highly spotted mates. Hence, we explored whether SOP values showed signs of sexual mating preferences in either female or male choice or both. We conducted a univariate general linear model (GLM) using SOP values as the dependent variable, sex as factor, and size of focal and stimulus fish as covariates since the size is an important variable that may influence mate choice in poeciliids. In many studies, male and female preferences for larger mating partners have been documented (Becker et al., 2012).

In addition to the GLM using SOP values as dependent variable, we also corroborated focal fish preferences for less or more spotted stimuli using paired t‐tests considering total association time with both types of stimuli in the two mating situations (male and females) as the dependent variable. As a *post‐hoc* procedure, we then performed Bayes Factor analysis in R (R Core Team 2014) using the BayesFactor R package (Morey et al., 2018) to estimate whether negative results were likely due to a true absence of a preference or whether the results are inconclusive. Bayes factor analyses compute Bayes factors, using Bayesian inference, to compare multiple mixed models. The Bayes factor quantifies the statistical support for or against the null hypothesis.

Furthermore, we used a linear regression to model the relationship between fish size and spotted area. In addition, we calculated the Pearson correlation coefficient to determine any significant correlation between the two variables.

#### 2.4.2 Color polymorphism in natural populations

We evaluated the prevalence of spotted morphs in natural populations from habitats with different salinity levels. We used Chi‐square tests to determine whether the frequency of spotted and nonspotted individuals was dependent on the type of habitat or related to sex.

Moreover, we measured the total body area covered by spots using ImageJ (same approach described for the sexual selection experiment) and compared the total body area covered by black spots of individuals collected in brackish/saltwater versus freshwater habitats using a Mann–Whitney U test after checking that the assumptions of homogeneity of variances and normality were violated through Levene's and Shapiro–Wilk's tests, respectively. All analyses were performed in SPSS version 26.

## 3 RESULTS

### 3.1 Sexual selection: Mating preferences relative to spotted area

**FIGURE 4:**
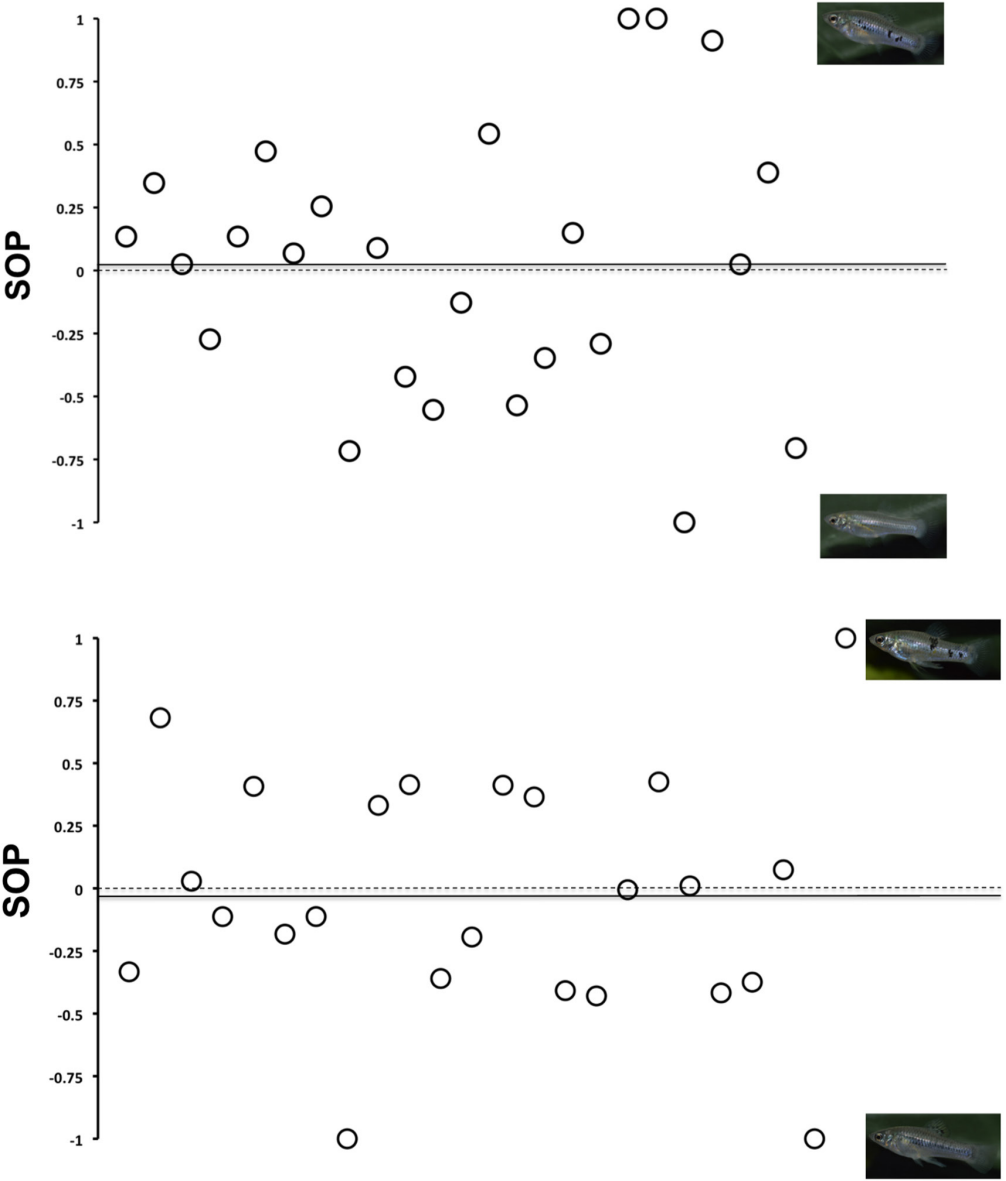
Distribution of individual strength of preference (SOP) values that resulted from the dichotomous association preference tests. Solid lines in the panels (in both cases close to the dashed lines representing the SOP value equal 0) correspond to the mean SOP value for males (top) and females (bottom).

Neither females nor males showed a mating preference for high‐ or low‐spotted mates. SOP values for both sexes ranged from −1.00 to 1.00, which suggests no clear mating preference relative to the total body spotted area. For males, the mean SOP value was 0.02 ± 0.53 (mean ± SE), and for females, the mean SOP value was −0.03 ± 0.48 (mean ± SE) (Figure 4). Overall, our GLM detected no significant difference between males and females for SOP values, F (1, 45) = 0.019, *p* = .890. In addition, we detected no significant effects of the size of the focal fish (F (1, 45) = 2.242, *p* = .125), size of the low‐spotted stimulus (F (2, 44) = 0.276, *p* = .602) and size of the high‐spotted stimulus (F (2, 44) = 0.190, *p* = .665). As is typical for livebearing fishes, focal females were on average larger than males (t = 12.923, *p* < .001), (female size [mean ± SE: 35.17 ± 4.19]; male size [mean ± SE: 21.68 ± 3.05]).

Because we did not find a significant preference, we used a Bayes factor analysis as a *post‐hoc* test to ask whether the absence of a preference is likely due to a true absence of a preference or not. To prepare for that, we used simple paired t‐tests on association time to confirm that neither males nor females preferred more or less spotted individuals as mates. Focal females spent roughly equal time with low‐spotted (mean ± SE: 220.75 ± 130.50 s) and high‐spotted stimulus males (mean ± SE: 201.25 ± 111.45 s) (t (23) = 0.50, *p* = .62). Similarly, we did not find differences in the association time of males with low‐spotted (mean ± SE: 251.84 ± 145.76 s) or high‐spotted females (mean ± SE: 267.28 ± 153.04 s) (t (24) = −0.26, *p* = .79).

Consequently, after the *t*‐tests, we ran a Bayes factor analysis (BFA) as a *post‐hoc* analysis to assess the two statistical models used in our traditional, Fisherian statistical test. In this analysis, the null hypothesis of an absence of preference is evaluated against the alternative hypothesis of the presence of preference, and the Bayes factor is used to quantify the support for or against the null hypothesis. The BFA for the female preference data showed moderate support for a lack of preference, suggesting that females actually do not possess a precopulatory preference (BFA = ±1.94%). In the case of males, the full model had strong support, which suggests that the factors influencing male association time are interacting (BFA = ±2.99%). This means that our results are inconclusive, and we cannot say definitively that there is no preference for female body spotted areas in male *L. vittata*.

**FIGURE 5:**
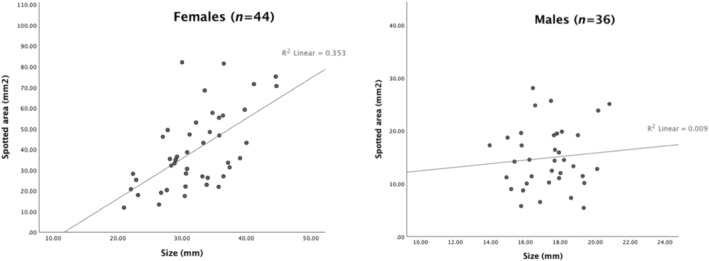
Representation of the linear regression analysis between the size of the fish and spotted area. Females (left panel) showed a positive, strong association between the size of the fish and spotted area (R^2^ = 0.353) while males (right panel) exhibited a positive, weak association between the two variables (R^2^ = 0.009).

Finally, we tested for a correlation between fish size and spotted area. The two variables were found to be significantly correlated in females (r (43) = 0.594, *p* < .001) but not in males (r (35) = 0.094, *p* = .587) (Figure 5).

In summary, there is no support for a role of sexual selection in maintaining the polymorphism for spots in *L. vitatta*.

### 3.2 Natural selection: Prevalence of black‐spotted morphs in natural populations of *L. vittata*

**FIGURE 6:**
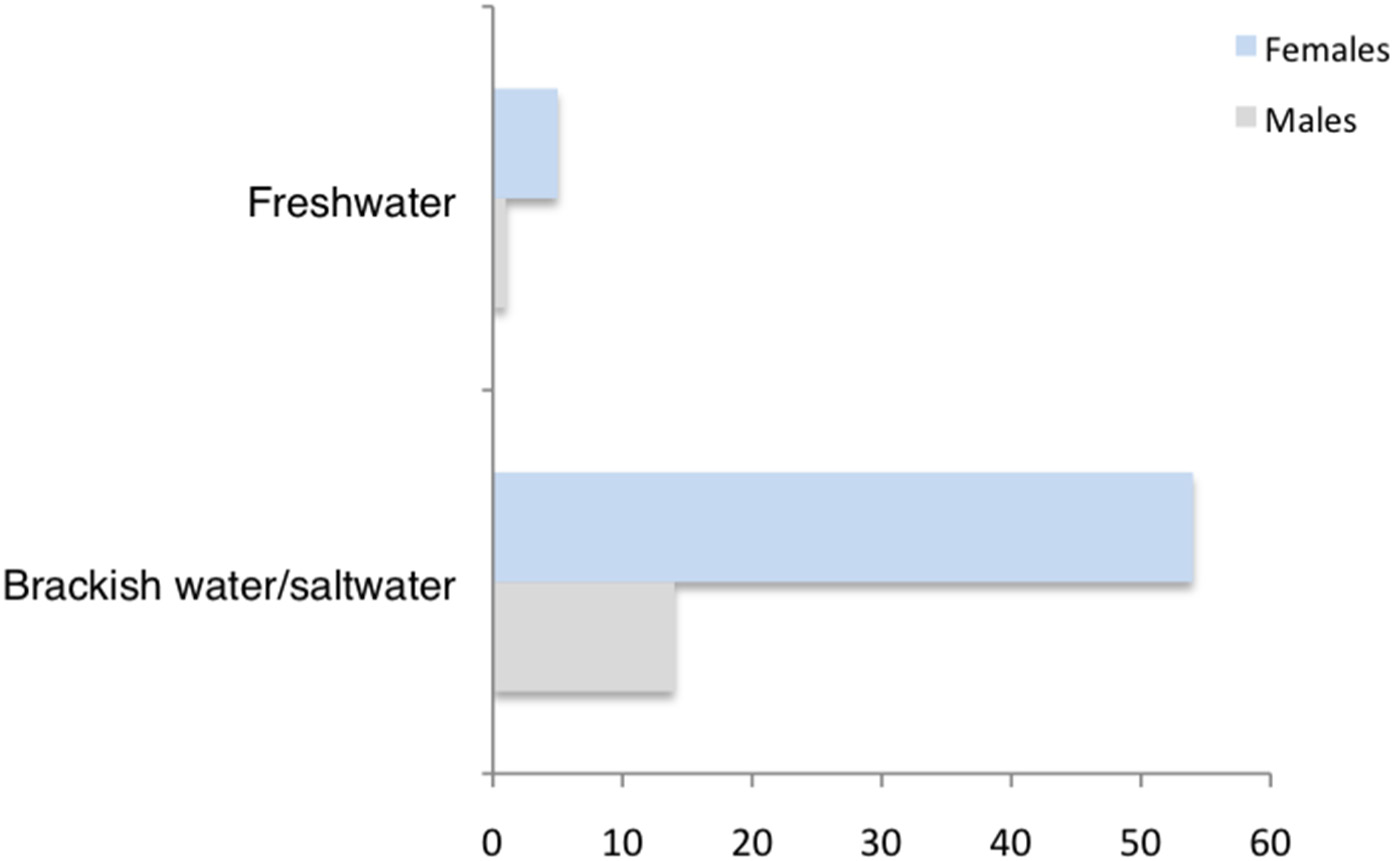
Frequency of spotted morphs in natural populations of *L. vittata* according to the type of habitat.

The type of habitat did not have an effect on the frequency of spotted and nonspotted individuals by sex within each habitat type: X^2^ (1, *N* = 111) = 1.2087, *p* = .271 (freshwater habitats) and X^2^ (1, *N* = 419) = 0.7794, *p* = .377 (brackish/saltwater habitats). However, we found that the frequency of black‐spotted morphs in natural populations of *L. vittata* changed between habitats. The frequency of spotted individuals was significantly higher in brackish and saltwater environments compared with freshwater habitats, where they were rare or completely absent in some localities (X^2^ (1, *N* = 529) = 9.9984, *p* = .001) (Figure 6).

In addition, we compared the degree of pigmentation of individuals (measured as total body area covered by black spots) collected in these different habitats. We detected no significant differences in the spotted area between individuals of brackish/saltwater (*N* = 68) and freshwater (*N* = 5) populations (U = 106, *p* = .177).

## 4 DISCUSSION

The results of our study support natural selection generated by variation in habitat salinity, rather than sexual selection, as the likely mechanism that explains the presence of color polymorphism in *L. vittata*. It has been shown that characteristics of the physical environment where fish species live can play an important role in changing the frequencies of melanistic morphs temporally and spatially (Horth & Travis, 2002; Zerulla & Stoddard, 2021). For instance, in the Green Swordtail (*Xiphophorus hellerii*) and the Atlantic Molly (*Poecilia mexicana*) spotted individuals are thought to have a social advantage for mating in low visibility conditions where spotted individuals are thought to be more visible than unspotted fishes (Culumber et al., 2014; Franck et al., 2001). However, it is not clear how salinity favors melanin spotting in *L*. *vitatta* as the effect of this and other physical factors (e.g., temperature, oxygen concentration, light exposure) on pattern expression and morph fitness remain unexplored for livebearing fishes (Meyer et al., 2006; Petrescu‐Mag et al., 2008). Our comparison between the spotted area of individuals collected in brackish/saltwater and freshwater habitats showed no differences in spotting patterns, which reinforces the idea of natural selection as more important for the higher frequency of spotted individuals in saline environments via a physiological factor linked to salinity in melanin spotting expression.

The combination of two different lines of evidence such as the assessment of mate choice preferences and collection of ecological data from the field offers a unique, interdisciplinary approach to explain the causes of color polymorphism. However, we have to acknowledge several limitations of both approaches. In the sexual selection experiment, we only analyzed individuals from one population in the case of females and two populations in the case of males. In particular for males, even though we did not find preferences for female spotting, the Bayes factor analysis showed that our results were inconclusive, and we could not say definitively that there was no preference for female body spotted area. This may be because we analyzed spotted males from two different populations or due to the fact that female spotted area was associated with female size while males exhibited no significant association between the two variables.

It has been shown that most of the short‐term experiments that assess how sexual selection acts in maintaining polymorphism are performed under simplified environments that constrain the interpretation of results. This is because mating preferences can be context dependent where social and ecological conditions, time and interpopulation variation can influence the outcome of each study (Alonzo & Sinervo, 2001; Rolán‐Alvarez et al., 1999). Conversely, long‐term studies that incorporate analyses of social effects in mating behavior and/or the quantification of different phenotype frequencies in natural habitats have been used as an alternative to avoid the limitations mentioned above (Culumber et al., 2013; Hurtado‐Gonzalez & Uy, 2010; Schartl et al., 1993). Regarding the field component of our study, one caveat is that the locations were sampled only once, and the collection times are far apart, which may not offer a complete view of the dynamic and temporal variation of color polymorphism, as well as changes in salinity levels in natural environments.

While our findings provide no clear support for a role of sexual selection, our results offer compelling evidence that connects the prevalence of spotted morphs in wild populations of *L. vittata* with the salinity of the habitat, suggesting that color polymorphism could be maintained in this species either directly or indirectly by natural selection. According to our results, the positive association between melanin spotting and moderate to high salinity levels may be a result of negative frequency‐dependent selection where the rare color variants (black‐spotted individuals in this case) are favored or have a selective advantage over the most common color morph (nonspotted individuals) (Ayala & Campbell, 1974; Chesson, 2000; Wellenreuther et al., 2014). The mechanism that may lead to this advantage is unclear, but we propose the following: Coastal habitats show spatially highly variable physical and chemical features since their position at the boundaries of terrestrial and marine ecosystems creates a patchwork of environmental gradients (Namba et al., 2020; Prado et al., 2014). This environmental heterogeneity inherent to coastal ecosystems leads to high levels of biodiversity (Palmer et al., 2011). Populations of *L. vittata*, a primarily detrivorous species, that live in coastal habitats can benefit from high productivity and food availability (Barus et al., 1980; Ponce de León & Rodriguez, 2010; Rodriguez‐Silva et al., 2021). However, higher productivity and the connectedness of coastal ecosystems also bring an increased predation threat by some diadromous and marine fishes that are known as major predators of livebearing fishes; for example, the bigmouth sleeper, *Gobiomorus dormitor* (Bacheler et al., 2004; Langerhans et al., 2007). Hence, *L. vittata* populations that live in estuarine habitats are likely to face higher predation pressure compared with populations that occur in purely freshwater environments suggesting that a tradeoff between productivity/food availability and predation may be responsible for maintaining the polymorphism. Specifically, highly spotted individuals could be positively selected in saltwater environments since a spotted color pattern may offer better camouflage in habitats with high levels of tannins dissolved in water and also abundant organic matter (mostly leaves) deposited on the bottom.

If our interpretation is correct, our findings would be in line with data from other studies. Predation pressure can have strong effects on the composition and structure of aquatic communities (Jackson et al., 2001; Matthews, 1998). It is also known to be a key selective agent facilitating speciation and favoring particular phenotypes and adaptive behaviors in natural populations. This has been thoroughly tested using both laboratory and field studies in the Trinidadian guppy (*Poecilia reticulata*) from low and high predation environments, where changes in life history traits, morphology, and behavior have been reported (Broder & Angeloni, 2014; Fraser & Gilliam, 1987; Kelley & Magurran, 2003; Kolluru et al., 2015; Magurran, 2005; Reznick et al., 1997; Reznick et al., 2008; Reznick & Endler, 1982). Contrasting predation pressure has been shown to be related to divergence in body shape coupled with assortative mating in *Gambusia* fishes from the Bahamas (Langerhans et al., 2007). Particularly in melanic side‐spotting poeciliid species, studies have found that predators prefer unspotted over spotted morphs, which may contribute to a selective survival advantage for melanic side‐spotted individuals (Zerulla & Stoddard, 2021). This pattern has been shown in populations of spotted Green Swordtail (*Xiphophorus hellerii*) where the predator species *Belonesox belizanus* ate more frequently unspotted than spotted specimens (Franck et al., 2001). Likewise, it has been described that unspotted individuals of the poeciliid species *Gambusia holbrooki* are more likely to be eaten by sunfishes (genus *Lepomis*) than unspotted morphs (Martin, 1977, 1986).

Other empirical experiments have also demonstrated, not only in fishes but also in other vertebrates, the significance of color patterns; especially background matching coloration (e.g., crypsis), in evading detection by predators (Gray & McKinnon, 2007). For instance, color polymorphism is maintained by selective predation in the case of the mottled rock rattlesnake (*Crotalus lepidus lepidus*) where individuals with color patterns contrasting with the substrate were more vulnerable to avian attacks (Farallo & Forstner, 2012). The analysis of three lizard species from a white sand ecotone (*Holbrookia maculata*, *Sceloporus undulatus*, and *Aspidoscelis inornata*) also demonstrates that phenotypic variation in color is quite common in heterogeneous habitats and substrates, which contributes to maintain color polymorphism through selection by differential predation (Rosenblum, 2006). There are also examples on how background matching and spatiotemporal variation could be implicated in maintaining polymorphism in poeciliids. In *Poecilia parae*, for instance, changes in the visual background resulted in variations in which different male morphs were most conspicuous to females across multiple localities. Also, the most colorful morphs were the most conspicuous morphs to predators (Hurtado‐Gonzalez et al., 2014). However, in the specific case of melanic side‐spotting pattern background matching has not been thoroughly studied (Zerulla & Stoddard, 2021).

Our findings are important to understanding the mechanisms that can potentially explain color polymorphism in species naturally occurring across highly heterogeneous environments such as the case of *L. vittata* that lives in freshwater and saline habitats. We provide novel evidence on how a measurable variable like salinity level influences polymorphism. In the case of our studied system, it seems that this variable is indirectly responsible for maintaining different color morphs likely due to the regulatory effect on predation regimes across saline gradients.

## AUTHOR CONTRIBUTIONS

**Rodet Rodriguez Silva:** Conceptualization (equal); data curation (lead); formal analysis (equal); funding acquisition (equal); investigation (equal); methodology (equal); writing – original draft (lead); writing – review and editing (equal). **Montrai Spikes:** Conceptualization (equal); formal analysis (equal); investigation (equal); methodology (equal); visualization (equal); writing – review and editing (equal). **Manuel Iturriaga:** Conceptualization (equal); data curation (equal); formal analysis (equal); investigation (equal); methodology (equal); visualization (equal); writing – review and editing (equal). **Ingo Schlupp:** Conceptualization (equal); data curation (supporting); formal analysis (equal); funding acquisition (supporting); investigation (equal); methodology (equal); supervision (lead); writing – review and editing (equal).

## FUNDING INFORMATION

Caribaea Initiative and National Geographic Society provided funding for our fieldwork.

## CONFLICTS OF INTEREST

The authors declare that they have no conflicts of interest.

### OPEN RESEARCH BADGES

This article has earned an Open Data badge for making publicly available the digitally‐shareable data necessary to reproduce the reported results. The data is available at [[insert provided URL from Open Research Disclosure Form]].

## Data Availability

Photos of specimens and data from the sexual selection experiment will be available in Dryad after publication.
