# Corrigendum

**DOI:** 10.1002/ece3.9984

**Published:** 2023-04-07

In the recent article by Rodriguez‐Silva et al., (2023), the authors would like to declare the financial support they received from the University of Oklahoma Libraries. The correct funding information is shown below:

**Funding information**

Caribaea Initiative; National Geographic Society; Financial support was provided by the University of Oklahoma Libraries' Open Access Fund.

The online version has been corrected.
